# Supplementary material for: Auditory sensory memory span for duration is severely curtailed in females with Rett syndrome
Source: Transl Psychiatry. 2019 Apr 9;9:130. doi: 10.1038/s41398-019-0463-0 (PMC6456588; doi:10.1038/s41398-019-0463-0)
Supplement: Supplementary file 1 — Supplementary information [file 41398_2019_463_MOESM1_ESM.docx]

**Supplementary Figures**


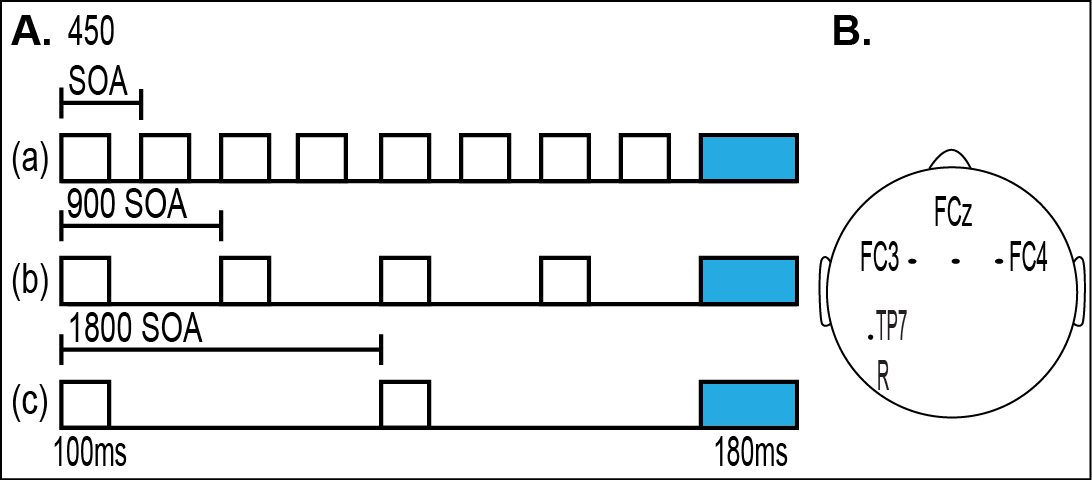


**Supplementary Figure 1:** Oddball paradigm design. A. Three experimental conditions were implemented, in which auditory stimuli were presented with varied stimulus onset asynchronies (SOAs): (a) 450 ms intervals between stimuli, (b) 900 ms interval between stimuli and (c) 1800 ms intervals between stimuli. B. The electrode sites of interest (FC3, FCz, FC4) and reference electrode R (TP7) are shown on a head map.

**Supplementary Tables**

**Supplementary Table 1.** Clinical demographics of all enrolled participants with Rett syndrome. Data from all participants in the shaded box were excluded from EEG analysis. RSSS = Rett Syndrome Severity Score; Seizure OS = Seizure on set; Age of R = Age of regression in months (mos.); N/A = Not Available.

| **Subject** | **Age** | **Mutations** | **(RSSS)** | **Seizures** | **Seizures OS** | **Ambulatory** | **Medications** | **Age of R** |
| --- | --- | --- | --- | --- | --- | --- | --- | --- |
| 1 | 8 | R255X | 13 | Yes | 48 mos | No | Trazadone, Depakote | N/A |
| 2 | 8 | R133C | 8 | Yes | 72 mos | Yes | Lovastatin, Topomax, Abilify, Lexapro, Depakene, Nexium | 21 mos |
| 3 | 15 | R306C | 7 | Yes | 168 mos | Yes | Lovastatin, Ambien, Trazodone | 50-60 mos |
| 5 | 14 | C964C | 5 | No | No | Yes | Trazadone | 156 mos |
| 7 | 8 | Deletion | 9 | No | No | Yes | Lovastatin | 11 mos |
| 8 | 10 | R255X | 6 | No | No | Yes | Lovastatin | 12 mos |
| 9 | 18 | R270X | 14 | N/A | N/A | No | none | N/A |
| 13 | 13 | Deletion | 14 | Yes | 30 mos | No | Depakene, Lexapro,Lactulose | N/A |
| 14 | 10 | Q170x mutation | 10 | N/A | N/A | Yes | N/A | 18 mos |
| 15 | 14 | Deletion | 12 | No | 72 mos | Yes | Risperdol and Necon | 144 mos |
| 16 | 22 | R133C | 12 | Yes | 108 mos | Yes | Depakote, Carintor, Artane, Copaxone | 17 mos |
| 17 | 10 | Deletion | 11 | N/A | N/A | No | Depakene | N/A |
| 18 | 17 | R270X | 13 | No | N/A | No | N/A | N/A |
| 20 | 17 | 418ins4 | 15 | Yes | N/A | No | Diastat, Depakene,  Carnitor | N/A |
| 21 | 20 | T158M | 11 | Yes | 72 mos | Yes | Lexapro, Lamictal | 15-18 mos |
| 22 | 6 | Large deletion | 12 | Yes | 60 mos | No | Valproic acid | N/A |
| 24 | 6.7 | Deletion exon 3&4 | 9 | Yes | 36 mos | No | Keppra, deplane | N/A |
| 25 | 13.2 | T158M | 12 | No | N/A | No | Nanadol, prevacid | 24 mos |
| 4 | 17 | T158M | 11 | Yes | 120 mos | Yes | Copaxone, Topomax, Lovastatin, | 10 mos |
| 6 | 7 | R294X | 7 | No | No | Yes | Lexapro, Lovastatin, Prilosec | 17 mos |
| 10 | 12 | R306C | 9 | No | No | Yes | Lexapro | 18 mos |
| 11 | 15 | R306C | 10 | No | No | Yes | Lexapro, Copaxone, Trazodone | N/A |
| 12 | 9 | Deletion | 7 | Yes | 24 mos | No | Depakene, Lactulose, Lovastatin | 30 mos |
| 19 | 7 | R133C | 10 | Yes | 42 mos | Yes | Abilify, Lexapro, Desyrel | 24 mos |
| 23 | 8.6 | R294X | 7 | No | N/A | Yes | Lexapro  Multivitamin | 24-29 mos |

**Supplementary Table 2.** Numbers of trial included in the analysis across condition in controls and in participants with Rett syndrome.

|  | TD (n)=27 | | | RETT (n)=18 | | | TD All Accepted | RETT All Accepted |
| --- | --- | --- | --- | --- | --- | --- | --- | --- |
|  | **450 SOA** | **900 SOA** | **1800 SOA** | **450 SOA** | **900 SOA** | **1800 SOA** | **Across Conditions** | **Across Conditions** |
| Avg. Accepted  Standard trials ± SD | 784.3  ±54 | 763.1  ±80 | 737  ±133 | 530.7  ±191 | 527  ±210 | 470.7  ±216 | 2284.3  ±184 | 1528.4  ±577 |
| Avg. Accepted Deviant trials ± SD | 137.9  ±54 | 136.6  ±14 | 128.9  ±23 | 91.5  ±32 | 95  ±38 | 81.9  ±39 | 403.5  ±34 | 268.4  ±101 |
